# Congenital Diaphragmatic Hernia and Iron Deficiency Anemia

**DOI:** 10.4274/Tjh.2013.0059

**Published:** 2013-06-05

**Authors:** Şinasi Özsoylu

**Affiliations:** 1 Fatih University School of Medicine, Department of Hematology, Ankara, Turkey

## TO THE EDITOR

The case described by Dr. Sarper and her colleagues in their letter to the editor, entitled “Severe iron deficiency anemia due to late presentation of congenital diaphragmatic hernia in a toddler”, was not a late but rather a delayed diagnosis case since iron deficiency anemia was diagnosed in this patient at least a year earlier with intermittent vomiting [1]. The diagnosis of a diaphragmatic hernia might be delayed up to at least 5 years of age as reported by us. This entity should not be extremely rare, since at least 9 patients in an 8-year period were seen by us [2]. We suggest that it should be looked for especially in very severe cases of iron deficiency anemia with frequent vomiting. Although restricted transfusion (<7 g/dL) was recommended in adult patients with upper gastrointestinal bleeding [3], as in the authors’ patients very severe anemia (<4 g/dL) was detected in 7 of our 9 patients.

## REPLY

In this case report [4], the title was “Late presentation of congenital diaphragmatic hernia….’’ because it is known that over 90% of the patients will be diagnosed either antenatally or will present with respiratory distress in the few hours of life [5]. The patient’s admission to our Center was at the age of 3 and she had a one-year history of intermittent vomitting and iron deficiency anemia. Özsoylu suggests that iron deficiency anemia due to diaphragmatic hernia is not very rare. Congenital diaphragmatic hernia occurs in about 1 in 3000 births [5]. It is not clear how many of these cases present with iron deficiency anemia. Similar to our message in the last paragraph, Özsoylu also emphasizes that in patients with very severe iron deficiency anemia and frequent vomiting diaphragma hernia must be considered in the differential diagnosis. We did not have the chance to reach Yetgin et al.’s paper as it is not present online [6].
